# The Relationship Between Orthodontic Malocclusion and Morphological Features of Sella Turcica Bridging and Ponticulus Posticus

**DOI:** 10.3390/medicina60111853

**Published:** 2024-11-11

**Authors:** Roberta Lekavičiūtė, Diana Šopagienė, Dainius Razukevičius, Giedrė Trakinienė, Kristina Lopatienė

**Affiliations:** 1 Faculty of Odontology, Lithuanian University of Health Sciences, J. Lukšos-Daumanto Str. 2, LT-50106 Kaunas, Lithuania; 2 Department of Radiology, Lithuanian University of Health Sciences, Eivenių Str. 2, LT-50161 Kaunas, Lithuania; diana.sopagiene@lsmu.lt; 3 Department of Oral and Maxillofacial Surgery, Lithuanian University of Health Sciences, Eivenių Str. 2, LT-50161 Kaunas, Lithuania; dainius.razukevicius@lsmu.lt; 4 Department of Orthodontics, Lithuanian University of Health Sciences, J. Lukšos-Daumanto Str. 6, LT-50106 Kaunas, Lithuania; giedre.trakiniene@lsmu.lt (G.T.); kristina.lopatiene@lsmu.lt (K.L.)

**Keywords:** ponticulus posticus, atlas, sella turcica bridging, lateral cephalogram, orthodontic malocclusions

## Abstract

*Background and Objectives*: Recent years have been marked by a search for new relationships between malocclusions and the morphological features of the cranial base and upper cervical spine. The aim of this study was to evaluate the relationship between skeletal orthodontic malocclusions and the morphological features of sella turcica bridging (STB) and ponticulus posticus (PP). *Materials and Methods*: The study sample consisted of 300 randomly selected pre-orthodontic treatment patients aged 7–40 years. Cephalometric analysis was performed twice by two researchers. The patients were divided into three groups according to the type of skeletal malocclusion. Statistical analysis was performed using SPSS 29.0 software. *Results*: The prevalence of incomplete PP was 40%, and the prevalence of complete PP was 11.33% and was significantly more common in males (56.7%). STB had a prevalence of 52.67% (15.67% complete, 37.0% partial), with a significantly higher prevalence in females (60.1%, *p* < 0.001). PP and STB were more common in Class II patients, followed by Class I and Class III. However, only PP showed a statistically significant difference (*p* = 0.042). According to logistic regression, men were twice as likely to have an open groove of PP (*p* = 0.015), while females had higher odds of partial and complete STB (*p* < 0.05). Class II malocclusion increases the odds of type 2 and type 4 PP as well as partial STB. However, it was not statistically significant (*p* > 0.05). *Conclusions*: The morphological features of sella turcica bridging and ponticulus posticus were related to sex and the orthodontic skeletal pattern. Ponticulus posticus was more prevalent in males, while sella turcica bridging was more prevalent in females; both conditions were more common in patients with a Class II skeletal pattern. Males were more likely to have an open groove of ponticulus posticus, whereas females were twice as likely to have both forms of sella turcica bridging.

## 1. Introduction

Malocclusion is one of the most prevalent oral health problems, following caries and periodontal disease. Its prevalence varies between 39% and 93% [1]. The development of malocclusions is influenced by genetic predisposition, environmental conditions, and ethnic factors [2]. Skeletal development of the neck and shoulders, the sella turcica (ST), and dental epithelial cells share a common embryonic origin [3,4]. Therefore, skeletal orthodontic anomalies can be associated with anomalies like sella turcica bridging or ponticulus posticus, which can cause clinical problems [5]. 

Research trials investigating the etiopathogenesis of various health problems, including cervical pain, hearing loss, symptoms of vertebrobasilar insufficiency (such as headache, diplopia, vertigo, dysarthria, and dysphagia), the Barré–Lieou syndrome, and photophobia, are receiving increasing attention in the scientific community [6,7,8,9,10]. There is a hypothesis that these health conditions could be linked to the relatively common atlanto-occipital anomaly identified as ponticulus posticus. It is alternatively referred to as the arcuate foramen, Kimerle anomaly or retroarticular vertebral artery ring [11]. Its prevalence varies from 1% to 46% [10,11,12,13]. Shahidi et al. and Lombardo et al. reported that PP is also associated with recurrent throat pain, foreign body sensation, otalgia, tinnitus, globus sensation, pain on neck rotation, dizziness, or even cerebral ischemia [10,14]. Wight et al. found that the bony ring was significantly more prevalent in chiropractic patients experiencing head pain without aura (i.e., without visual or auditory disturbances) [15]. 

Ponticulus posticus is a malformed bridge located at the posterior part of the superior articular process and the posterolateral part of the superior margin of the posterior arch of the atlas [11,16]. It either fully or partially surrounds the sulcus, enabling the vertebral artery (VA) to travel from the transverse foramen into the foramen magnum [7]. Therefore, this pathology can be classified into two types: partial and complete [17], and it can also be unilateral or bilateral [18]. After the formation of complete PP, movement of the VA may be restricted, leading to reduced blood flow to the brainstem, cerebellum, and occipital cortex. According to a study by Lo Giudice et al., this compression can result in health problems such as unexplained neck pain and headaches [8]. In addition to the VA, PP includes the suboccipital nerve and is connected to the dura by the atlanto-occipital membrane [7]. 

The relationship between the morphological characteristics of the sella turcica and various health problems is an area of ongoing research. Studies have demonstrated associations between ST morphology and a range of health conditions. Identification of these morphological features can aid in the diagnosis and management of several syndromes and provides insights into the genetic mechanisms underlying craniofacial development [19,20]. Roomaney et al. found that patients with genetic disorders such as Down, Marfan, or Turner syndromes exhibit variations in ST morphology (size, shape, or increased bridging), while sella turcica bridging (STB) is more frequently observed in individuals with severe occlusal anomalies or other craniofacial malformations [20,21]. 

The sella turcica is a saddle-shaped bony structure found on the intracranial surface of the body of the sphenoid bone [4,22,23]. It contains important nervous, endocrine, vascular, and bony structures, making it a region of significant clinical interest across various medical fields, including endocrinology and neurology. From front to back, it consists of two anterior clinoid processes, the tuberculum sellae, which is the pituitary fossa covered with diaphragma sellae, and two posterior clinoid processes [4,22]. STB is a variation characterized by the calcification of the ligament connecting the anterior and posterior clinoid processes of the sphenoid bone, which can lead to the fusion of these processes [3,4,24]. Its prevalence varies from 1.1% to 13% [25]. Dadgar et al. in their study detected that abnormal embryogenic development of the sphenoid bone may also contribute to the formation of this anomalous bridge [24]. 

Both sella turcica bridging and ponticulus posticus may have clinical implications in orthopedic and neurological settings. Identifying the PP is crucial for the surgical treatment of atlantoaxial instability, as it may create a misleading intraoperative impression of a wide posterior arch, potentially leading to injury of the vertebral artery during screw insertion into the lateral mass of the atlas [8]. When inserting the atlas lateral mass screw through the posterior arch into the lateral mass, the PP may be mistaken for a thickened posterior arch. This misunderstanding could result in the surgeon drilling the borehole too high, which may lead to iatrogenic injury to the third segment of the vertebral artery [16]. The sella turcica also has clinical relevance in surgical procedures. The morphology of the sella turcica should be carefully evaluated during surgical planning, as Cuschieri et al. concluded that abnormalities in ST can complicate surgical procedures, further increasing the difficulty of exposing the cavernous sinus due to the complex neurovascular connections in the region [21].

Routine orthodontic examination includes both clinical and radiological assessments, with a lateral cephalometric radiograph (LCR) performed as part of the radiological evaluation. During cephalometric analysis, the sella turcica is used to assess the relationships between the maxilla, the mandible, and the cranial base [3]. Even though the sella turcica is routinely used in cephalometric analyses, the morphology of the sella turcica, including potential pathologies such as sella turcica bridging, is rarely evaluated. These anatomical structures are easily identified in lateral cephalometric radiographs, and their evaluation is one of the most important parts in orthodontic treatment planning [26]. Besides assessing skeletal maturation and malocclusions, they are useful in the diagnosis of a variety of conditions and anomalies related to the craniofacial and cervical vertebral areas [27,28,29,30,31,32,33]. LCRs provide diagnostic information about the skull, face, and the upper cervical spine, especially when dental and craniofacial abnormalities are present [5,6,26,27]. The radiographic anatomy of the cervical spine region and its potential relationship with certain pathologies has not received sufficient attention [6]. Orthodontists should identify these conditions during routine orthodontic radiographic examinations because many of these anomalies remain asymptomatic until adolescence or early adulthood. This would prevent potential degenerative conditions and significant complications in the future. The aim of this study was to evaluate the relationship between skeletal orthodontic malocclusions and the morphological features of sella turcica bridging and ponticulus posticus.

## 2. Materials and Methods

This retrospective study was registered and approved by the Kaunas Regional Biomedical Research Ethics Committee, with the registration number BE-2-71. The study consisted of 300 patients (153 females and 147 males) randomly selected from the database of the Department of Orthodontics at the Lithuanian University of Health Sciences. The age range was 7–40 years (mean age, 15.10 years, SD, 4.84 years). LCRs of the included patients were used to evaluate skeletal pattern, incidence of PP, and STB. The required sample size was determined to be 300 based on the sample size calculation using the Krejcie and Morgan sample size determination table. The inclusion criteria were the following: healthy patients, with no previous orthodontic treatment or surgery, patients who had a high-quality pre-treatment lateral cephalometric radiograph of the head, showing the first four cervical vertebrae and clear imaging of the sella turcica. The exclusion criteria were the following: patients with syndromic conditions or other developmental abnormalities, patients with missing or low-quality pre-treatment lateral cephalometric radiographs, not showing the first four cervical vertebrae and sella turcica.

LCRs were analyzed according to a standardized protocol by a senior dental student and an expert orthodontist, under the supervision of a radiologist. Before the study, the observers were instructed and a pilot study of 20 LCRs was performed by two observers under supervision of an expert radiologist. The presence and degree of STB and PP were assessed twice for every patient using LCRs. In addition, 20 randomly selected LCRs were repeatedly examined by the same observers after two weeks and Cohen’s Kappa analysis was performed to determine the agreement among both investigators. The agreement was considered substantial, with a weighted kappa of 0.72.

Cephalometric analysis was performed using WebCeph software (version 1.5.0). The ANB (A-point—Nasion—B-point) angle was evaluated according to Steiner’s analysis [34]. Based on the type of skeletal malocclusion, subjects were categorized into three skeletal pattern groups: Class I (ANB angle 0–4°) (*n* = 113), Class II (ANB angle > 4°) (*n* = 155), and Class III (ANB angle < 0°) (*n* = 32). 

**Figure 1:**
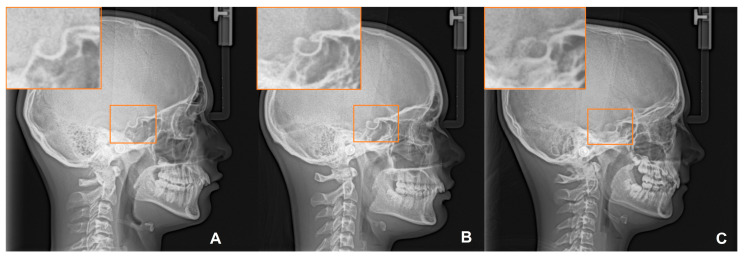
The classification of sella turcica bridging: Type I (**A**); Type II (**B**); Type III (**C**).

Sella turcica analysis was performed according to the standardized scoring scale proposed by Leonardi et al. [35], and the degree of STB was classified into three types (Figure 1): Type 1 (no calcification): the length of the sella turcica is equal to or greater than 3/4 of the diameter; Type 2 (partial calcification): the length of the sella turcica is less than or equal to 3/4 of sella diameter; Type 3 (complete calcification): only the diaphragma sellae is visible on the radiograph.

**Figure 2:**
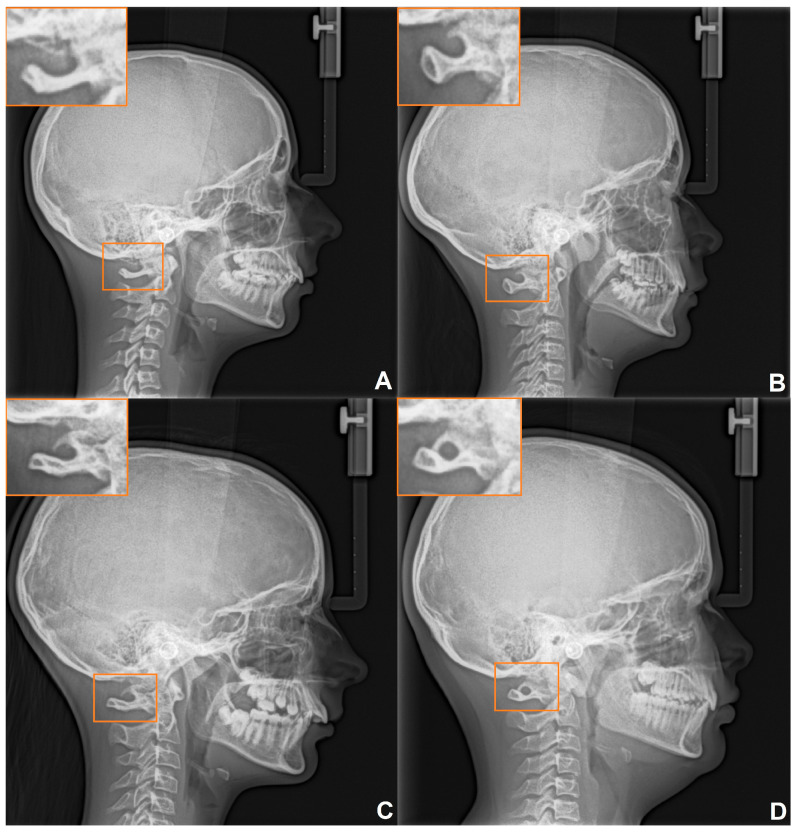
The classification of ponticulus posticus: Type I (**A**); Type II (**B**); Type III (**C**); Type IV (**D**).

The measurement of ponticulus posticus was determined by evaluating the LCRs, considering its nominal measurement scale with the following types (Figure 2): Type 1 (no calcification): no mineralization is observed in the atlanto-occipital ligament; Type 2 (an open groove): partial calcification extending from the lateral mass beyond 180°, without reaching the posteromedial margin of the vertebral artery groove; Type 3 (a half-open groove): partial calcification extending from the lateral mass beyond 270°, without reaching the posteromedial margin of the vertebral artery groove; Type 4 (complete calcification, a full ring): complete bone bridge involving the atlanto-occipital ligament, with complete mineralization extending from the lateral mass to the posteromedial margin of the vertebral artery groove.

### Statistical Analysis

Statistical Package for the Social Sciences (IBM SPSS Statistics © Version 29.0.0.0) software was used for statistical analysis. Associations between qualitative independent variables (age group, sex, and skeletal malocclusions) and qualitative outcome variables (the presence of sella turcica bridging and ponticulus posticus development) were examined using the chi-square test, the difference in frequency being expressed as a z-score. The Kolmogorov–Smirnov test was used to assess the normality of quantitative variables. The Kruskal–Wallis H test was used to assess the association between non-normally distributed qualitative variables (age) across different outcome groups. Multinomial logistic regression was used to quantify the influence of predictors (qualitative independent variables (age group, sex, and skeletal malocclusions)) and outcome variables. Odds ratios (OR) were used to express the change in odds of being in a non-reference group compared to the reference group. Goodness-of-fit was expressed as the Nagelkerke coefficient. Odds ratios and 95% confidence intervals were determined. Results with *p*-values of 0.05 or less were considered statistically significant.

## 3. Results

In this study, 300 lateral cephalograms were evaluated. Based on their age, patients were divided into three groups: 10 years and under (*n* = 27), 11–14 years (*n* = 135), 15–18 years (*n* = 97), and 19 years and older (*n* = 41). Descriptive statistics for skeletal malocclusion classes within the groups are presented in Table 1.

Ponticulus posticus (types 2, 3, and 4 combined) was identified in 157 patients (52.33%). Among the entire patient group, 143 (47.67%) did not have PP, 89 (29.67%) had type 2, 34 (11.33%) had type 3, and type 4 was observed in 34 patients (11.33%). PP was statistically significantly more common in males (*p* = 0.005), while females had a significantly higher prevalence of the non-calcified type (*p* = 0.05). According to the type of malocclusion, PP was significantly more frequent in patients with Class II malocclusion (*p* = 0.042) (Table 2).

Sella turcica bridging (types 2 and 3 combined) was found in 158 patients (52.67%). Of these, 111 patients (37.0%) had partial bridging, and 47 patients (15.67%) had complete bridging, while 142 patients (47.33%) had a normal sella with no bridging. In females, the incidence of STB was significantly higher (*p* < 0.001). Additionally, partial bridging was significantly more frequent in females, while the absence of bridging was significantly more common in males (*p* = 0.004) (Table 3).

**Figure 3:**
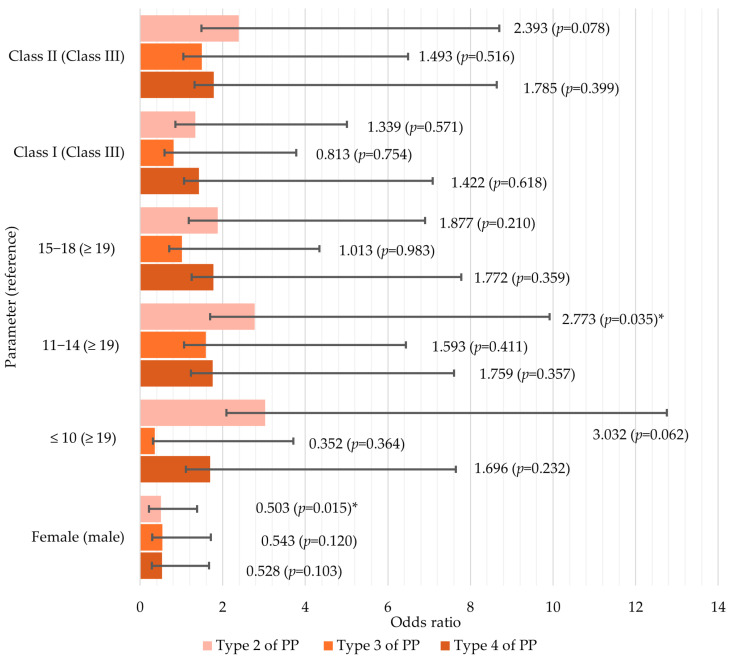
Logistic regression analysis of the relationship between age, sex, skeletal malocclusion class, and ponticulus posticus (* *p* ≤ 0.05).

The association between these factors and PP is presented in Figure 3. The reference category was type 1 of ponticulus posticus. The model fit was statistically significant (*p* = 0.024), although none of the independent variables significantly improved it. However, the contributions of sex and age groups were close to statistical significance (*p* = 0.055 and *p* = 0.051, respectively). Overall, the model explained 11% of the variance in PP.

Sex and age were associated with the presence of PP. Males were twice more likely to have type 2 rather than type 1 PP, with the difference being statistically significant (*p* = 0.015). Additionally, the likelihood of females having type 3 or type 4 PP was also lower (OR = 0.5 for both PP types). However, this difference was not statistically significant (*p* > 0.05). In relation to age, the odds of having type 2 PP significantly increased about 3 times for individuals aged 11–14 years (OR = 2.8, *p* = 0.035). Also, the presence of Class II skeletal pattern would increase the odds of having type 2 (OR = 2.4) and type 4 (OR = 1.8) PP by about 2 times. However, no significant differences were observed between skeletal pattern groups and PP (*p* > 0.05).

**Figure 4:**
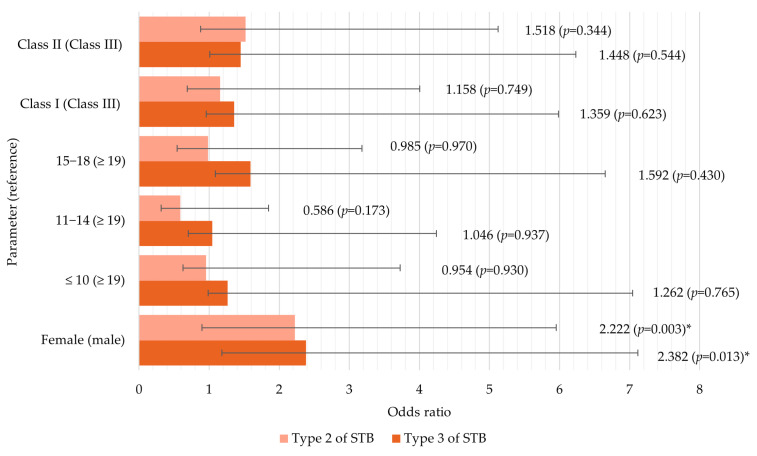
Logistic regression analysis of the association between age, sex, skeletal malocclusion, and sella turcica bridging (* *p* ≤ 0.05).

The association between age, sex, the type of malocclusion, and STB is presented in Figure 4. The reference category was type 1 of sella turcica bridging. The independent variables included in the regression model did not significantly predict the type of STB (*p* = 0.132). According to Nagelkerke’s R^2^, the model explains approximately 6.5% of the variance in the outcome.

Only sex was significantly associated with the presence of STB. Females had approximately 2 times higher odds of having both partial (OR = 2.2) and complete (OR = 2.4) STB (*p* = 0.003 and *p* = 0.013, respectively). Patients aged 11–14 were less likely to have partial STB (OR = 0.6), while those aged 15–18 were more likely to have complete STB (OR = 1.6). The presence of a Class II skeletal pattern can predict a higher likelihood of partial STB (OR = 1.5), while Class I is associated with an increased probability of complete STB (OR = 1.6). However, no significant differences were observed between age or malocclusion groups and STB (*p* > 0.05).

## 4. Discussion

There is widespread and growing interest in factors influencing the presence of ponticulus posticus or sella turcica bridging. Scientific research on the relationship between these pathologies and various orthodontic anomalies is still in progress, and analyses evaluating the etiopathogenesis of these conditions are being performed. Given the ongoing discussions on this topic, our study aimed to investigate the relationship between ponticulus posticus and sella turcica bridging with sex, age, and skeletal malocclusions using lateral cephalograms from the Lithuanian population. Understanding the presence of these structures is important, as they may be linked to the patients’ overall health and should be closely examined during routine radiographic evaluations. 

The etiology of ponticulus posticus is unknown; it could be hereditary or age-related, possibly due to degenerative changes associated with aging [10,36]. On the other hand, PP originates from the activity of neural crest cells during fetal development, contributing significantly to the formation of the skull, vertebral column, maxilla, mandible, and dental tissues [37]. Furthermore, abnormalities in the cervical vertebrae have a relationship with malformations in craniofacial structures, condyles, jaws, and occlusion [24]. Additionally, ponticulus posticus has a relationship with dental anomalies such as hyperdontia, primary molar ankylosis, and missing maxillary lateral incisors [24,38]. Kaya et al. found that partial calcification of PP was higher in subjects with palatally impacted canines, tooth transposition, and third molar agenesis (TMA), while full calcification was more frequent only in subjects with TMA [4]. 

Dental abnormalities have been related to variations in the ST, and this relationship can be due to their shared embryogenic origin, which involves the neural crest cells responsible for forming structures like the ST and teeth. This association may potentially be explained by genetic changes that impact the development of the teeth, the ST, and the midface [24]. The anterior and posterior walls of the sella turcica have different embryological origins: while the posterior wall develops like vertebrae influenced by the notochord, the anterior wall forms from neural crest cells [4]. They play a role in the development of the neck and shoulders, as well as in the calcification of the sella turcica and the formation and eruption of teeth [4,5]. Anomalies affecting the neural tube may increase the risk of abnormal development in the skull, vertebral column, and teeth [37]. Therefore, dentoskeletal abnormalities, including malocclusions, may be associated with abnormalities in head and neck position, cervical inclination, and orthopedic problems [5,37]. Although STB has been linked to multiple hereditary developmental syndromes affecting the craniofacial region and various systemic disorders, it is also associated with local dental anomalies such as tooth transposition, hypodontia, missing mandibular second premolars [27], and dental aplasia [24]. Partial STB is more common in subjects with palatally impacted canines (PIC) and peg-shaped maxillary lateral incisors, while complete STB is more frequent in those with TMA [4]. However, Siddalingappa et al. reported that both forms of STB were more common in patients with dental anomalies compared to those without any anomalies [38]. 

Since there are ongoing discussions about orthodontic anomalies and their relationship to morphological and postural changes in the head and neck, we examined the relationship between sella turcica bridging and ponticulus posticus in different skeletal orthodontic malocclusions. Our findings revealed that PP was significantly more prevalent in males and in patients with a Class II skeletal pattern compared to those with Class I or Class III malocclusions. However, a similar study conducted by Adisen et al. found no significant difference in the prevalence of partial or complete PP types based on sex or the skeletal pattern [12]. Another study also reported no variation in the type or prevalence of PP across malocclusion groups, although PP was also more frequent in skeletal Class II patients [8]. The relationship between age and various types of PP and STB was also analyzed. In our study, PP was more common in patients aged 11 to 14 years, whereas STB was most frequently observed in patients aged 15 to 18 years. Lo Giudice in their study evaluated the relationship between PP and skeletal maturity and found that the mean age at which patients first exhibited PP was 10.4 years for the complete form and 9.3 years for the partial form, and the prevalence of both PP forms was higher in young patients [8]. Different results were reported by Atilla: the prevalence of PP and STB varied across different growth periods, and the prevalence of complete PP and complete STB increased from the preadolescent to the post-adolescent period [26]. 

In examining the prevalence of PP, our study revealed an overall rate of 52.33%, with complete PP present in 11.33% of cases. A few research studies were carried out to evaluate global variations in the prevalence of PP and STB. Cuschieri et al. analyzed global variations in the prevalence of sella turcica bridging. The findings showed that STB is the most prevalent in Europe, followed by Asia, the Americas, and Africa [21]. In the context of ethnic variability regarding the prevalence of ponticulus posticus, this condition is most prevalent in North American populations, while the lowest prevalence is observed in populations from India and South Korea [39]. In addition, data regarding the prevalence of PP in the Italian population are similar to those reported in Turkish studies [39]. Among young Italian patients, it was observed that 28.24% of the subjects exhibited PP, with 74.33% of these cases being bilateral and 25.67% unilateral [40]. These findings are consistent with our study, which also reported a higher prevalence of PP in males compared to females [40]. Comparatively, a study conducted on the American population reported a slightly lower overall PP prevalence of 12.6%, with the complete form at 8.7% and the partial form at 3.9%. In this population, males also showed a higher prevalence, and both partial and complete forms of PP were predominantly observed in individuals aged 7–13 years [8]. When considering the prevalence of PP by morphological classification, unilateral partial PP was the most common, followed by bilateral partial, unilateral complete, mixed, and bilateral complete [18]. In contrast, the Chinese population demonstrated the lowest overall prevalence of PP at 8.01%; however, it was still more prevalent in males than in females. Additionally, PP was more frequently observed on the left side than on the right [41]. Our study reported an STB prevalence of 52.67%, with both forms being more common in females. One of the studies analyzed prevalence in the Eastern Asia population and found the complete STB form in 6.6% of cases and the partial form in 56.9% of cases. Contrary to our findings, females more frequently exhibited the partial form, while males more commonly had the complete bridge [42]. The same authors conducted another study where they evaluated left- and right-side ST in cone-beam computed tomography (CBCT) images and found a higher prevalence of STB on the right side than on the left [43]. 

These findings emphasize the importance of these morphological features in orthodontic diagnosis and treatment planning. The presence of PP or STB can be assessed not only on LCRs but also with other radiological techniques, such as CBCT, which allows for more precise three-dimensional evaluations, but its high radiation exposure makes it less suitable for initial assessments [44]. In contrast, LCRs are more accessible, safer, and easier to evaluate [45]. During routine radiographic examinations, the presence of PP or STB can be easily identified and evaluated. Patients exhibiting morphological features of STB or PP during growth should be referred to a team of healthcare specialists for the development of an individualized treatment plan. This study has some limitations. In cephalometric analysis, it is difficult to determine the unilateral or bilateral presence of PP and STB because it cannot reconstruct their three-dimensional morphology, and while the sample size was sufficient to achieve significant results, a larger sample could improve the reliability of the findings. Continued research in this field is needed to further our understanding and improve clinical outcomes for patients affected by these pathologies.

## 5. Conclusions

The morphological features of sella turcica bridging and ponticulus posticus were significantly related to sex and the orthodontic skeletal pattern. Ponticulus posticus was significantly more common in males and in patients with a Class II skeletal pattern, while sella turcica bridging was significantly more prevalent in females. Females were twice as likely to have both forms of sella turcica bridging.

## Figures and Tables

**Table 1:** Descriptive statistics of the study population.

Groups	Subjects, *n*	Mean Age ± SD	Female, *n* (%)	Male, *n* (%)
Class I	113	14.87 **±** 4.47	67 (43.8)	46 (31.3)
Class II	155	15.19 **±** 5.06	74 (48.4)	81 (55.1)
Class III	32	15.47 **±** 5.12	12 (7.8)	20 (13.6)

SD: standard deviation.

**Table 2:** The prevalence and variations of ponticulus posticus in relation to age, sex, and skeletal malocclusion.

Group	Ponticulus Posticus			Type of Ponticulus Posticus				
	Present	Absent	*p* Value	Type 1	Type 2	Type 3	Type 4	*p* Value
	*n* (%)	*n* (%)		*n* (%)	*n* (%)	*n* (%)	*n* (%)	
Age								
≤10	13 (48.1)	14 (51.9)	0.235	14 (51.9)	12 (44.4)	1 (3.7)	0 (0.0)	0.171
11–14	78 (57.8)	57 (42.2)		57 (42.2)	44 (32.6)	18 (13.3)	16 (11.9)	
15–18	50 (51.5)	47 (48.5)		47 (48.5)	26 (26.8)	10 (10.3)	14 (14.4)	
≥19	16 (40.0)	24 (60.0)		24 (60.0)	7 (17.5)	5 (12.5)	4 (10.0)	
Sex								
Female	68 (43.3) *	85 (59.4) *	0.005	85 (55.6) *	38 (24.8)	15 (9.8)	15 (9.8)	0.05
Male	89 (56.7) *	58 (40.6) *		57 (39.0) *	51 (34.9)	19 (13.0)	19 (13.0)	
Skeletal malocclusion								
Class I	51 (32.5)	62 (43.4)	0.042	62 (43.4)	28 (31.5)	10 (29.4)	13 (38.2)	0.285
Class II	92 (58.6) *	63 (44.1)		63 (44.1)	54 (60.7)	20 (58.8)	18 (52.9)	
Class III	14 (8.9)	18 (12.6)		18 (12.6)	7 (7.9)	4 (11.8)	3 (8.8)	

* Statistical significance when *p* ≤ 0.05.

**Table 3:** The prevalence and variations of sella turcica bridging in relation to age, sex, and skeletal malocclusion.

Group	Sella Turcica Bridging			Type of Sella Turcica Bridging			
	Present	Absent	*p* Value	Type 1	Type 2	Type 3	*p* Value
	*n* (%)	*n* (%)		*n* (%)	*n* (%)	*n* (%)	
Age							
≤10	15 (9.5)	12 (8.5)	0.427	12 (8.5)	11 (9.9)	4 (8.5)	0.694
11–14	64 (40.5)	71 (50.0)		71 (50.0)	43 (38.7)	21 (44.7)	
15–18	56 (35.4)	41 (28.9)		41 (28.9)	39 (35.1)	17 (36.2)	
≥19	23 (14.6)	18 (12.7)		18 (12.7)	18 (16.2)	5 (10.6)	
Sex							
Female	95 (60.1) *	58 (40.8) *	<0.001	58 (40.8) *	66 (59.5) *	29 (61.7)	0.004
Male	63 (39.9) *	84 (59.2) *		84 (59.2) *	45 (40.5) *	18 (38.3)	
Skeletal malocclusion							
Class I	59 (37.3)	54 (38.0)	0.516	54 (38.0)	40 (36.0)	19 (40.4)	0.809
Class II	85 (53.8)	70 (49.3)		70 (49.3)	61 (55.0)	24 (51.1)	
Class III	14 (14.0)	18 (12.7)		18 (12.7)	10 (9.0)	4 (8.5)	

* Statistical significance when *p* ≤ 0.05.

## Data Availability

The datasets used and analyzed during the current study are available from the corresponding author on reasonable request.
